# Comparative Analysis of Components and Biological Activities in Different Parts of *Gastrodia elata* Blume

**DOI:** 10.3390/metabo16060406

**Published:** 2026-06-10

**Authors:** Yuru Zhang, Hong Zhang, Xue Meng, Yu Zhang, Di Wang, Juan Chen

**Affiliations:** Institute of Chinese Materia Medica, Shaanxi Academy of Traditional Chinese Medicine, Xi’an 710003, China; lycheezyr@163.com (Y.Z.);

**Keywords:** *Gastrodia elata* Blume, different organs, metabolomics, antioxidant activity, enzyme inhibition, multivariate statistical analysis

## Abstract

**Highlights:**

**What are the main findings?**

- A non-targeted UHPLC-MS/MS-based metabolomics approach was employed to compare metabolite characteristics across six parts of *Gastrodia elata* Blume (above-ground and underground tissues). Furthermore, correlation analysis was conducted on their in vitro biological activities and differential metabolites.
- *L*-arginine and *L*-aspartate are metabolites initially associated with the differential antioxidant and *α*-glucosidase inhibitory activities observed in stems and capsule shells.

**What are the implications of the main findings?**

- This study preliminarily provided metabolomics evidence for the difference between the above-ground and underground parts of *Gastrodia elata* Blume.
- This study provided a reference for fully utilizing the resources of *Gastrodia elata* Blume and improving plant utilization.

**Abstract:**

**Background:** *Gastrodia elata* Blume (GE) is a widely used plant with both medicinal and edible properties. Besides the underground tubers, its above-ground parts also have certain medicinal potential. However, most of these resources are frequently discarded during production and processing. The differences in chemical composition and bioactivity among different parts of GE remain unclear. **Methods:** In this research, the non-targeted metabolomics method was used to detect the metabolites in the stem (ST), capsule shell (CS), seed (SE), arrow end (AE), middle end (ME), and navel end (NE) of GE. Differential metabolites were screened by multivariate statistical analysis. The pathway enrichment analysis of differential metabolites was carried out by the Kyoto Encyclopedia of Genes and Genomes (KEGG) platform. The antioxidant and *α*-glucosidase inhibitory activities of extracts from six different parts of GE were determined in vitro, followed by correlation analysis between biological activities and key metabolites. **Results:** Metabolites in different parts of GE were significantly different, including lipids, organic acids, organoheterocyclic compounds, phenylpropanoids, benzenoids, and organic oxygen compounds. The characteristics of metabolites in the underground and above-ground parts of GE were clearly distinct. The contents of lipids, organoheterocyclic compounds, and organic acids were the highest in SE, SE and CS, ST, and AE, respectively. The KEGG pathway enrichment analysis preliminarily suggested that the differences in metabolites from different parts of GE might be related to the arginine biosynthesis pathway, which involves seven metabolites, including *L*-glutamic acid, *L*-arginine, *L*-ornithine, and *L*-aspartate. Compared with the tuber (the conventional medicinal part), ST and CS exhibited excellent antioxidant properties and inhibitory activity against *α*-glucosidase. Correlation analysis indicated that *L*-arginine and *L*-aspartate were positively correlated with the biological activity of GE and may be components preliminarily associated with its difference in activity. **Conclusions:** This study provided preliminary comparative evidence for the metabolite characteristics from different parts of GE, thereby facilitating the further development and utilization of its above-ground resources.

## 1. Introduction

*Gastrodia elata* Blume (GE) is mainly distributed in the mountainous areas of East Asia, including China, South Korea, Japan, and India. In China, GE, as a traditional precious medicinal herb, has been included in the list of “A material traditionally used as both food and medicine”, providing policy support for the development of related health products of GE [1]. GE has the effects of calming the liver, suppressing wind, and stopping spasms. It is mainly used in clinical practice to treat conditions such as headache, dizziness, and epilepsy. GE mainly contains phenolic glycosides, sugars and their glycosides, steroids, organic acids and their esters, amino acids and their derivatives, and other chemical components [2,3]. Modern pharmacological studies have shown that GE has significant pharmacological effects in protecting the nervous system, inducing sedation and hypnosis, combating depression, antioxidation, and immune regulation [4,5]. There are approximately 120 kinds of health foods made of GE and its extracts on the market, which have multiple health functions such as improving sleep, enhancing immunity, and maintaining blood pressure health [6]. Researchers have detected a variety of essential nutrients and trace elements, such as Ca, Fe, and Mg, from GE [7]. Furthermore, in traditional medicinal cuisine, GE is often combined with ingredients such as chicken and fish for dietary therapy to treat headaches and dizziness. With the formal inclusion of GE in the homology catalog of medicine and food, it has broad prospects for development and application in the fields of new tea drinks, functional foods, and health drinks. It can be seen that GE has high medicinal and nutritional value.

Due to the scarcity of wild resources of GE and the high demand for both food and medicine, it has been included in the list of wild plant protection in China. In order to meet the needs of human beings, most of the GE available in the market is mainly cultivated, and GE is mostly used as medicine in underground tubers. To assess the quality of medicinal materials more accurately, the tubers of GE are typically divided into AE, ME, and NE, mainly based on the external morphological characteristics of the herbs and the differential distribution of internal chemical components. The latent bud at the top of the tuber is an important symbol of the traditional identification of “Dongma”. ME is the main part of the tuber, with multiple concentric rings on its surface. NE has round umbilicus-shaped, sunken scars, which are round marks left by the separation of the tuber from the mother hemp or funiculus. Studies have shown that the stems of GE also have anti-convulsive and antibacterial effects. However, after the process of hybrid breeding, they are often discarded, resulting in a significant waste of resources. The aerial parts of GE, capsule shells, and seeds also contain effective components such as gastrodin, which has certain medicinal potential [8]. At present, there are few studies on the non-medicinal parts of GE, which limits the full utilization of its resources. Therefore, it is necessary to further compare and analyze the differences in chemical composition and efficacy between medicinal and non-medicinal parts of GE so as to provide a research basis for fully utilizing the above-ground parts of GE, alleviating the shortage of GE resources, and improving the utilization rate of GE plants.

At present, the analysis of chemical components in GE mainly focuses on the determination of the content and types of individual component groups, while the research on the overall metabolic components and the differences in components at different parts is not yet in-depth enough. Non-targeted metabolomics technology, characterized by high throughput, high sensitivity, and unbiasedness, has become an important tool for analyzing the differences in metabolites from different parts of traditional Chinese medicine. Metabolomics primarily involves the application of high-throughput screening techniques combined with chemometric methods to quantitatively and qualitatively analyze endogenous metabolites in organisms. Researchers have utilized non-targeted metabolomics technology to analyze the differential metabolites in different medicinal parts of plants, such as Aurantii Fructus Immatures [9], *Hemerocallis citrina* [10], and *Salsola collina* Pall [11]. Zhou, G. et al. [12] conducted a comparative metabolomics study on the chemical components and biological activities of GE at different growth stages. However, there have been no reports on the study of differential metabolites in different parts of GE. Considering the potential resource utilization value of the renewable plant parts of GE, in this study, we employed non-targeted UHPLC-MS/MS metabolomics analysis technology to systematically investigate the distribution characteristics of metabolites in the aerial and underground parts of GE. Principal component analysis (PCA) and orthogonal partial least squares discriminant analysis (OPLS-DA) were employed to screen out the differential metabolites in different parts of GE, and KEGG pathway enrichment analysis was used to elucidate the potential biosynthesis and regulatory mechanisms. In addition, correlation analysis was conducted on the antioxidant activity, *α*-glucosidase inhibitory activity, and key differential metabolites of extracts from different parts, revealing the organ-specific accumulation pattern of chemical components in GE. The research results provide a scientific theoretical basis for the screening of medicinal parts of GE, the targeted development of secondary metabolites, and quality control.

## 2. Materials and Methods

### 2.1. Materials and Reagents

**Figure 1:**
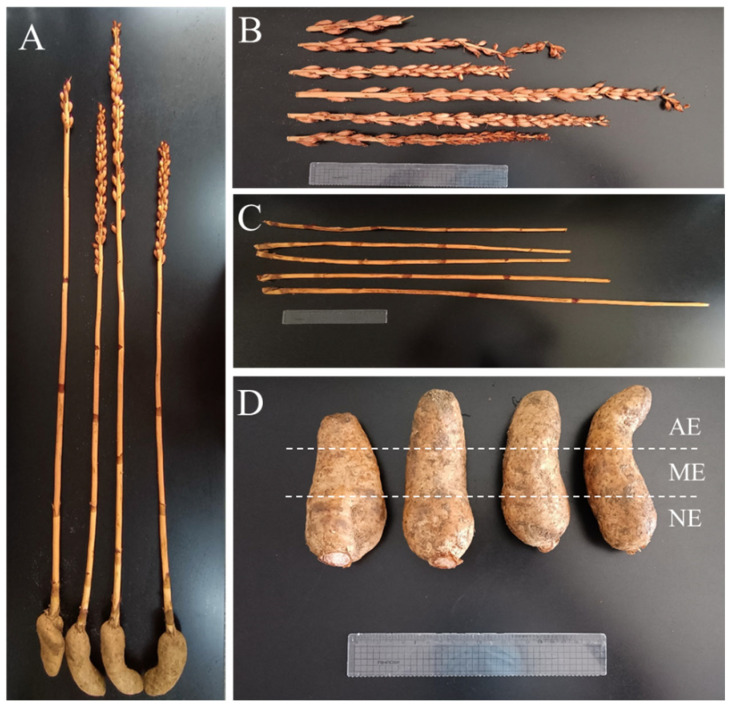
The morphological characteristics of GE. (**A**) The entire plant of GE; (**B**) inflorescence; (**C**) stem; (**D**) tuber.

The fresh specimens of GE were collected in their entirety from the GE medicinal plant cultivation base in Hanzhong City, Shaanxi Province, on 20 April 2025. The base is located in Sanchuanba Village, Wulongdong Town, Lueyang County, Hanzhong City, Shaanxi Province. Its coordinates are 106°11′55″ E, 33°31′20″ N, and the altitude is 1363 m. They were identified by Zhang Hong, a researcher from the Shaanxi Academy of Traditional Chinese Medicine, as the orchid family plant *Gastrodia elata* Blume. The GE was separated into six parts: SE, CS, ST, AE, ME, and NE. The various parts of GE were cleaned with flowing water until there was no sediment or other impurities on the surface of the sample (Figure 1). The fresh tubers of GE contain enzymes that can decompose gastrodin. Studies have shown that steaming can promote the transformation of chemical components in GE, inhibit enzymatic degradation, and also increase the content of gastrodin and crude polysaccharides [13,14]. Therefore, in this study, the treatment methods for the above-ground and underground parts of GE were distinguished. Each part of the tuber was steamed at 100 °C for half an hour and dried at 60 °C in an electrically heated constant-temperature forced-air drying oven. The other parts were dried directly. It should be noted that this different treatment of above-ground and underground parts may also lead to differences in their metabolites.

For UHPLC-Q Exactive Orbitrap-MS analysis, mass spectrometry grades of methanol, acetonitrile, and formic acid were purchased from Thermo Fisher (Waltham, MA, USA). Analytical grade methanol for sample extraction was obtained from Tianli Chemical Reagent Co., Ltd. (Tianjin, China). Diphenyl-1-picrylhydrazyl (DPPH, Batch No.: C16266521), 4-nitrophenyl-*α*-D-glucopyranoside (pNPG, Batch No.: C17611767), and acarbose (Acarbose, Batch No.: C17593788) were provided by MACKLIN Biochemical Technology Co., Ltd. (Shanghai, China). The 2,2-azino-bis (3-ethylbenzothiazoline-6-sulfonic acid) diammonium salt (ABTS) scavenging capacity kit (Batch No.: 20241211) was supplied by Jiancheng Bioengineering Institute (Nanjing, China). Vitamin C (VC, Batch No.: D01GB169601) and *α*-glucosidase (Batch No.: KS347846) were acquired from Shanghai Yuanye Biotechnology Co., Ltd. (Shanghai, China). PBS buffer (Batch No.: 28224968CJ) was purchased from Beijing Lanjieke Technology Co., Ltd. (Beijing, China). Sodium carbonate was purchased from Tianjin Baishi Chemical Co., Ltd. (Tianjin, China). Distilled water was purchased from Watson’s Group (Guangzhou, China).

### 2.2. Sample Solution Preparation

Ultrasound extraction is a widely used classical method in metabolomics, which has been proven to be capable of effectively extracting the main active components in GE and can cover the target metabolites in this study [15,16]. The samples from different parts of the GE were ground and passed through a 60-mesh sieve. Approximately 1.0 g of the powder was accurately weighed and extracted in 20 mL of an 80% methanol-water mixture for 30 min under ultrasonic conditions. The extract was centrifuged at 12,000 rpm for 10 min at 4 °C, and the supernatant was filtered through a 0.22 μm membrane. In order to ensure the consistency of sample sources in the metabolite identification and biological activity analysis experiments of GE, the prepared sample solutions were stored at 4 °C and divided into two portions, which were used respectively for subsequent UHPLC-Q Exactive Orbitrap-MS and bioactivity analysis. Six biological replicates were set for each group of samples. An equal volume of mixed sample solutions was used as quality control (QC) samples to balance the chromatography-mass spectrometry system and monitor the instrument status.

### 2.3. Chromatographic and Mass Spectrometry Conditions

UHPLC-MS/MS analyses were performed using a Vanquish UHPLC system (Thermo Fisher, Dreieich, Germany) coupled with an Orbitrap Q Exactive^TM^ HF mass spectrometer (Thermo Fisher, Erlangen, Germany) in Novogene Co., Ltd. (Beijing, China). In total, 2 μL of samples were injected into a Hypersil Gold C18 column (1.9 μm, 2.1 mm × 100 mm) using a 12 min linear gradient at a flow rate of 0.2 mL/min. The column temperature was set to 40 °C. The mobile phase consisted of (solvent A) 0.1% formic acid-water solution and (solvent B) methanol. The elution gradient was as follows: 0~1.5 min, 2% B; 1.5~3 min, 2~85% B; 3~10 min, 85~100% B; 10~10.1 min, 100~2% B; 10.1~12 min, 2% B.

Mass spectrometry scanning was performed in positive and negative ion modes with a mass scan range of 100–1500 Da. The parameters of the ESI source were set as follows: spray Voltage: 3.5 kV; sheath gas flow rate: 35 psi; aux Gas flow rate: 10 L/min; capillary temperature: 320 °C; S-lens RF level: 60; aux gas heater temperature: 350 °C; MS/MS secondary scan was data-dependent scans. The parameters of the primary and secondary scanning were set as follows, respectively: resolution: 60,000 FWHM and 15,000 FWHM; automatic gain control (AGC) target: 3,000,000 and 200,000; and the maximum injection time was maintained at 100 ms and 25 ms. The normalized collision energy was 20, 40, and 60 eV; de-clustering voltage: 3.2 V; iontophoresis RF level (S-lens RF level): 60; the temperature of the sample automatic injector: 4 °C. Throughout the entire analysis batch, a QC was injected after every 6 samples were analyzed to monitor the stability of the system.

### 2.4. Qualitative and Semi-Quantitative Analysis of Metabolites

The original data was converted into mzXML format by ProteoWizard. First, XCMS software version 3.7.0 (Novogene Co., Ltd., Beijing, China) was used for peak extraction and peak quantification. Peak alignment was performed for different samples by retention time, mass-to-charge ratio, and other parameters. Then, according to the information of mass error being less than ±5 ppm and adduct ions, the metabolites were analyzed by comparing with the self-built high-quality secondary spectrum database NovoMetDB, which was built by Novogene Co., Ltd. The metabolites were positively annotated by databases such as the KEGG database (https://www.genome.jp/kegg/pathway.html, accessed on 2 December 2025) and the LIPID Maps database (http://www.lipidmaps.org/, accessed on 2 December 2025). After eliminating the background ions according to the blank sample, the original quantitative results were standardized to obtain the relative peak area, and the relative content of the metabolites was calculated according to the mass spectrometry peak area. The compounds with a coefficient of variation in relative peak area greater than 30% in QC samples were deleted. After screening based on the coefficient of variation, approximately 64% of the features were retained for subsequent analysis (Table S1). Finally, the annotation and relative quantitative results of metabolites were obtained. The data processing part of metabolomics was accomplished based on the Linux operating system (version 6.6 of CentOS) and R software (version 3.4.3, https://www.r-project.org/, accessed on 15 December 2025).

### 2.5. Multivariate Statistical Analysis

PCA was performed using the pcaMethods package in R software version 3.4.3, with autoscaling and 2 principal components retained. OPLS-DA analysis was performed using the R software version 3.4.3 of the ropls package. Because the number of biological replicates of the sample was greater than 3, we used 7-fold cross-validation to evaluate the performance of the model to calculate the R^2^Y and Q^2^ values. The reliability of the model was verified by 200 permutation tests. The higher the variable importance in the projection (VIP) value, the greater the contribution of the metabolites to the model classification. VIP > 1 was considered an important contributing factor. The fold change (FC) reflects the changes in the expression level of metabolites between the two groups. The FC values of metabolites between the two groups were calculated by the OPLS-DA model, and the statistical significance of each metabolite between the two groups was calculated according to the *t*-test. The p.adjust package was used to perform FDR correction on the *p*-value. In this study, VIP ≥ 1, FC ≥ 2, or FC ≤ 0.5 and adjusted *p*-value (adj *p*) < 0.05 were used as the criteria to screen differential metabolites, and the volcanic map and cluster heat map were drawn with the ggplot2 package and Pheatmap package of R software version 3.4.3, respectively. In the hierarchical clustering analysis (HCA), the standardization method was scale, and bidirectional clustering was selected; that is, clustering was performed on both rows and columns. Finally, the differential metabolites were mapped to the KEGG pathway database (http://www.kegg.jp/kegg/pathway.html, accessed on 17 January 2026), and bubble plots were drawn using the ggplot2 package of R software version 3.4.3 for metabolic pathway enrichment analysis. When *p* < 0.05, the metabolic pathway was considered to be significantly enriched.

### 2.6. In Vitro Antioxidant Activity Assays

#### 2.6.1. DPPH Assay

According to the method reported in the literature, the DPPH free radical scavenging activity of extracts from six different parts of GE was analyzed [17,18]. An appropriate amount of DPPH was dissolved in anhydrous ethanol to prepare a DPPH solution with a mass concentration of 0.08 mg/mL. In total, 100 μL of GE sample solution with different mass concentrations and positive control VC reference solutions were taken and placed in the 96-well plate, respectively; 150 μL of DPPH solution was added to each well, mixed, incubated in a water bath at 37 °C for 30 min under dark conditions, and the absorbance *A*_1_ was measured at 517 nm by the enzyme detector (Thermo Fisher Scientific, Waltham, MA, USA). In the blank group, an 80% methanol-water solution was used to replace the test solution to measure the absorbance *A*_0_. In the control group, 80% methanol-water solution was used instead of the DPPH solution to determine the absorbance *A*_2_. Three duplicate wells were set for each group, and the average value was taken. The clearance rate can be calculated using the following equation [19]:

DPPH free radical clearance rate (%) = [1 − (*A*_1_ − *A*_2_)/*A*_0_] × 100%

The GraphPad software (GraphPad Prism 8.3.0, USA) was used to fit the logarithmic curve with the sample concentration (mg/mL) as the abscissa and the clearance rate as the ordinate. The fitting formula was obtained, and the half-maximal inhibitory concentration (IC_50_) was calculated. The IC_50_ values of antioxidant and *α*-glucosidase inhibitory activities in this study were calculated based on the total solid concentration.

#### 2.6.2. ABTS Assay

The determination method of ABTS was improved according to the method of Orcan, P. et al. [20]. The kit was equipped with 4 reagents for detection. Before the test, the enzyme solution was diluted 10 times with reagent 1 to form the reagent 4 application solution. The concentrated substrate was diluted 40 times with distilled water to prepare the reagent 3 application solution, and then the ABTS working solution was prepared according to the ratio of reagent 1: reagent 2: reagent 3 application solution = 76:5:4 and stored at room temperature in the dark. 10 μL of GE sample solution with different concentrations was added in sequence in a 96-well plate, followed by 20 μL of reagent 4 application solution, and finally, 170 μL of ABTS working solution was added. The reaction was carried out at room temperature in the dark for 6 min, and the absorbance *A*_1_ was measured at 405 nm. In the blank group, an 80% methanol-water solution was used to replace the test solution to measure the absorbance *A*_0_. In the control group, an 80% methanol-water solution was used instead of the ABTS free radical working solution, and its absorbance *A*_2_ was measured. Three replicates were set for each group, and the average value was taken. The scavenging rate was calculated according to the following formula:

ABTS free radical clearance rate (%) = [1 − (*A*_1_ − *A*_2_)/*A*_0_] × 100%

The calculation method for the IC_50_ value was the same as “2.6.1”.

### 2.7. Inhibitory Activity Assay of α-Glucosidase

According to the description by TSAI et al. [21,22], the inhibitory activity of different parts of GE on *α*-glucosidase was determined. The GE extract and the positive control acarbose were diluted with phosphate-buffered saline (PBS, pH = 7.0) into different concentrations of solution for later use. In total, 25 μL of PBS buffer was used as the buffer system, and 25 μL of *α*-glucosidase (0.5 U/mL) and 25 μL of the sample solution to be tested were added. The mixture was shaken and reacted at 37 °C in a water bath for 15 min. After removal, 50 μL of p-NPG (10 mM) solution was added and mixed evenly. The reaction was then carried out at 37 °C in a water bath for another 15 min. After removal, 100 μL of sodium carbonate solution (0.1 mol/L) was added to terminate the reaction. The absorbance was measured at 405 nm. The PBS buffer instead of the sample was used as the blank control, the acarbose instead of the sample was used as the positive control, and the background group did not contain *α*-glucosidase. The sample group was labeled as *A*_1_, the background group as *A*_2_, and the blank group as *A*_3_. Three replicates were set for each group, and the average value was taken. The inhibition rate was calculated according to the following formula [23]:

*α*-glucosidase inhibition rate I (%) = [1 − (*A*_1_ − *A*_2_)/*A*_3_] × 100%

### 2.8. Data Analysis

All experiments were conducted independently, and the experimental data were presented as mean ± standard deviation (SD). The analysis and plotting were performed using OriginPro software (version 2024, OriginLab Corporation, Northampton, MA, USA). One-way analysis of variance (ANOVA) was used with IBM SPSS Statistics (version 27.0.1, Chicago, IL, USA) to test the differences between the groups of samples (*p* < 0.05). Pearson correlation analysis and plotting were conducted using GraphPad Prism 8 software (*p* < 0.05; two-tailed).

## 3. Results

### 3.1. Metabolic Spectrum Analysis of 6 Different Parts of GE

The original data of samples from SE, CS, ST, AE, ME, and NE of GE, as well as the QC samples, were logarithmically converted and standardized. Then, they were imported into R software version 3.4.3 for PCA and correlation analysis. As can be seen from Figure S1A, the distances between the three QC sample points were relatively close, and they were clustered at the origin. Additionally, the correlation graph of the QC samples (Figure S1B) was also an important reference indicator. The correlation between QC samples was greater than 0.95, indicating that the detection instrument ran stably, with good repeatability, small systematic error, and stable and reliable test data.

**Figure 2:**
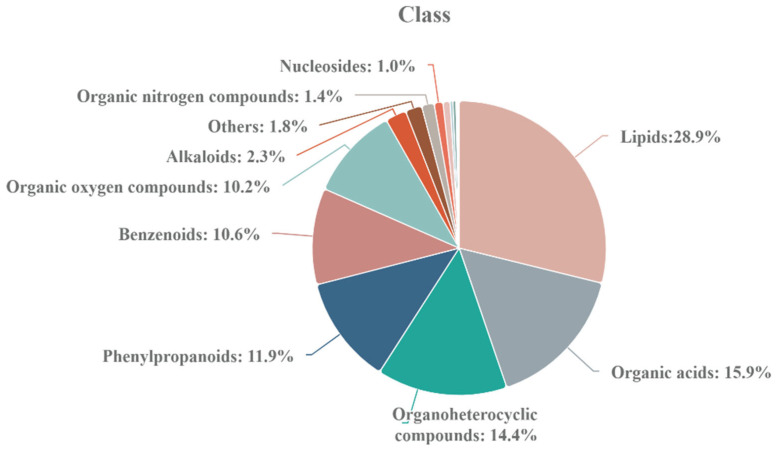
Metabolite classification pie chart.

The total ion chromatogram of a QC sample in positive and negative ion modes is shown in Figure S2. By matching the retention time, accurate molecular weight, and fragment ions of the metabolites with the local database, 2472 compounds were initially inferred from the six parts of GE, among which 1351 were compounds in the ESI^+^ mode, and 1121 were compounds in the ESI^−^ mode. Of the 2472 metabolites identified, about 45% of the metabolites were mapped to the KEGG database, and the remaining metabolites were supplemented with annotations by databases such as LIPID Maps and PubChem. These metabolites can be classified into 18 types, including 714 lipids, 393 organic acids, 355 organoheterocyclic compounds, 294 phenylpropanoids, 262 benzenoids, 251 organic oxygen compounds, etc. (Figure 2, Table 1). The detailed data of all positively annotated metabolites in GE were presented in Table S2.

### 3.2. Distribution of Metabolites in Different Parts of GE

**Figure 3:**
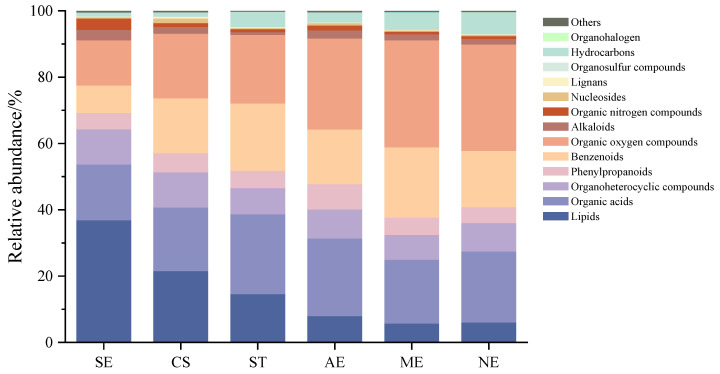
Distribution of the components in different parts of GE.

The distribution of metabolites is closely related to the organs of plants. To study the differences in the metabolic profiles in six different parts of GE, the accumulation bar charts of metabolites in different parts were drawn based on the relative peak areas of the metabolites, as shown in Figure 3. Lipids, organic acids, organoheterocyclic compounds, phenylpropanoids, benzenoids, and organic oxygen compounds were the main components of GE. The relative content of different metabolites in different parts of GE was different, and the difference was significant (Table S3). The lipid content in SE was significantly higher than that in other parts, followed by CS and ST. In addition, the content of organoheterocyclic compounds in SE and CS was significantly higher than that in other parts, and there was no significant difference between them. The distribution of organic acids was the highest in ST and AE, and there was no significant difference between them, followed by NE and ME. Moreover, the content of phenylpropanoids was the highest in AE. Compared with other parts, the content of organic oxygen compounds in the three parts of AE, ME, and NE was significantly higher. Additionally, the content of benzenoids in ME was the most abundant. It could be seen that the distribution trends of different components in different parts of GE were different, which may be related to its special functions in plant metabolism. For instance, the glycerolipids in lipids mainly serve as energy reserves, while glycerophospholipids are the main components of cell membranes and also serve as metabolic fuels and signaling molecules, participating in the process of plants’ defense against pathogens [24]. Studies have shown that the heterocyclic compounds in GE have certain activities in anti-tumor and the treatment of neurological diseases. Abdel-Salam, O.M. et al. [25] found that citric acid can reduce oxidative stress injury in the brain and has a neuroprotective function. Phenylpropanoids have an important influence on the antioxidant and disease resistance of plants. The accumulation of total phenols, flavonoids, and lignin in fresh GE can enhance its antioxidant capacity and reduce oxidative damage [26]. The representative components of organic oxygen compounds, such as gastrodin and parishin, can protect and regenerate the nervous system through various mechanisms [27,28,29].

### 3.3. Multivariate Statistical Results

**Figure 4:**
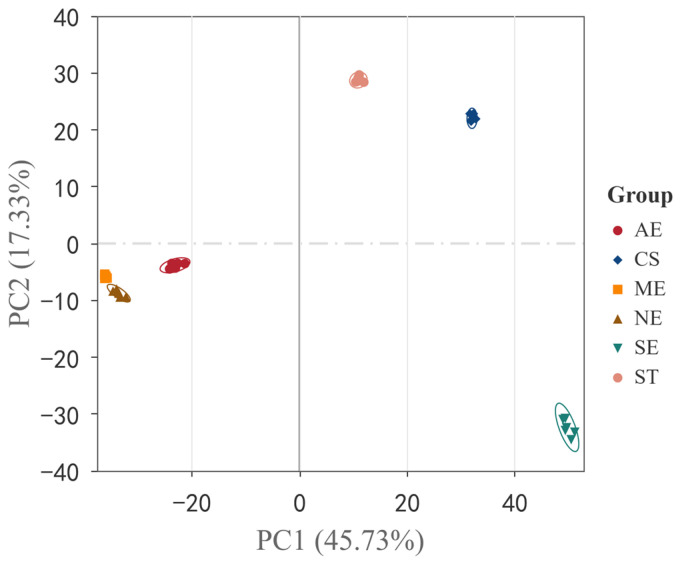
PCA score plots in different parts of GE.

Multivariate statistical methods were employed to analyze the identified metabolite data in order to distinguish the different parts of the GE samples. Firstly, an unsupervised PCA was conducted on all the samples, resulting in a multivariate data matrix. The PCA score plot is shown in Figure 4. Each point represented a separate sample. The spatial distribution of the samples within each group was relatively concentrated, indicating good repeatability of the samples within the group; the spatial distribution of the samples between groups was relatively dispersed, indicating significant differences in the characteristics of metabolites among different parts. The first two principal components accounted for 63.06% of the total variance in the PCA graph. The tuber of GE, as an important medicinal part, had significantly different chemical components from SE, CS, and ST (the above-ground parts). However, the three parts of AE, ME, and NE had similar metabolic characteristics. In summary, the metabolic profiles in six different parts of GE showed certain differences and similarities.

**Figure 5:**
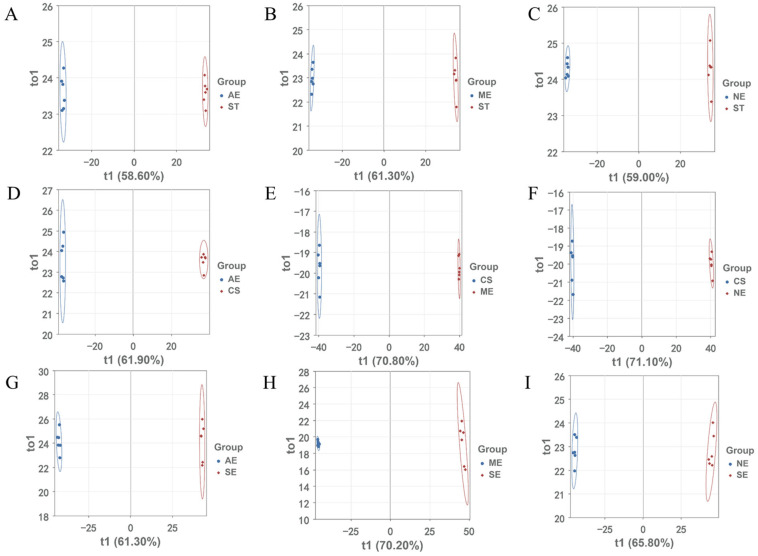
OPLS-DA score plots of OPLS-DA model plots for the comparison groups, ST vs. AE, ST vs. ME, ST vs. NE, CS vs. AE, CS vs. ME, CS vs. NE, SE vs. AE, SE vs. ME, and SE vs. NE (**A**–**I**).

PCA only reduces dimensions based on the variance structure and does not consider grouping. It may overlook key but low-abundance differential metabolites and cannot be directly used for screening differential metabolites [30]. HCA can only display clustering distances and cannot quantify the contribution size of each variable (metabolite) to classification. In contrast, supervised OPLS-DA usually sets VIP > 1 as the threshold for screening key differential metabolites and can filter out orthogonal noise to maximize the inter-group differences [31]. To further clarify the metabolite differences between the medicinal parts and non-medicinal aerial parts of GE, based on the PCA results, supervised OPLS-DA was performed on nine pairs of plant tissues, and the score plots are shown in Figure 5. Table S4 summarizes the parameters of the OPLS-DA model. The closer R^2^X, R^2^Y, and Q^2^ are to 1, the higher the stability and reliability of the model, indicating good reproducibility and predictive ability [32]. It is worth noting that the high R^2^ and Q^2^ in the OPLS-DA model may be due to the large biological differences between groups or the relatively small sample size. At the same time, the established OPLS-DA model was verified by the Permutations test (*n* = 200). The results were shown in Figure S3, and the arrangement values of R^2^ and Q^2^ were all less than the original values. The R^2^ values exceeded the Q^2^ values, and the intercept of the ordinate of the regression line of Q^2^ was negative, indicating that the permutation test did not indicate obvious overfitting, but supervised models should be interpreted cautiously [33]. The model was stable, but attention should still be paid to the risk of overfitting that may exist in small sample data.

### 3.4. Comparative Analysis of Differential Metabolites in Different Parts of GE

**Figure 6:**
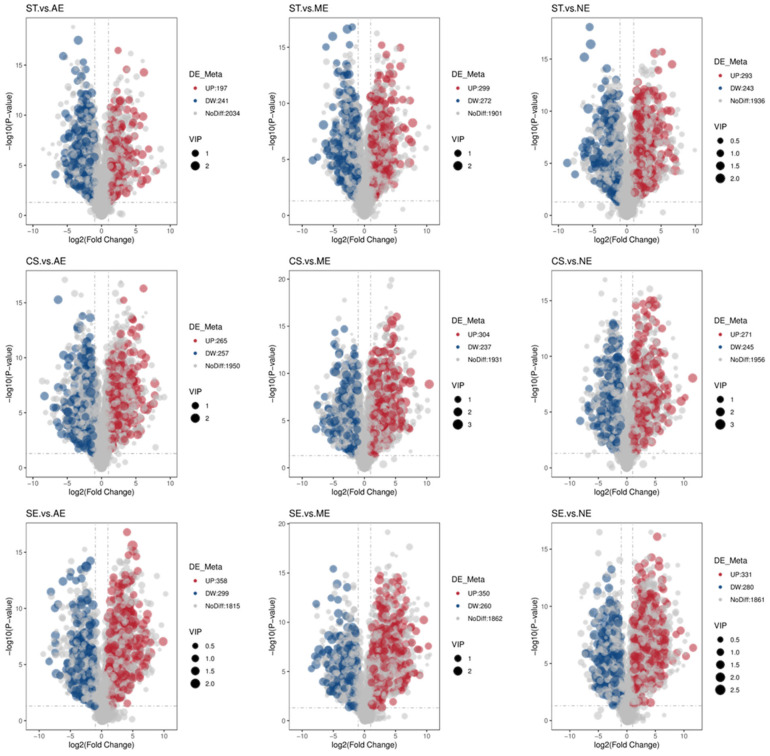
The volcano plots of differential metabolites in comparison groups. Red dots and blue dots represented up-regulated and down-regulated differential metabolites, respectively. Gray dots indicated that there was no significant change in metabolites.

In order to gain a deeper understanding of the differences in metabolites among different parts of GE, the differential metabolites were screened based on the criteria of FC ≥ 2 or ≤ 0.5, VIP ≥ 1, and adj *p* < 0.05. The screening results were presented in the form of a volcano plot (Figure 6). Compared with AE, 438, 522, and 657 metabolites were differentially accumulated in ST, CS, and SE, respectively, of which 197, 265, and 358 metabolites were up-regulated. Compared with ME, 571, 541, and 610 metabolites were differentially accumulated in ST, CS, and SE, respectively, of which 299, 304, and 350 metabolites were up-regulated. Compared with NE, 536, 516, and 611 metabolites were differentially accumulated in ST, CS, and SE, respectively, of which 293, 271, and 331 metabolites were up-regulated (Figure S4).

**Figure 7:**
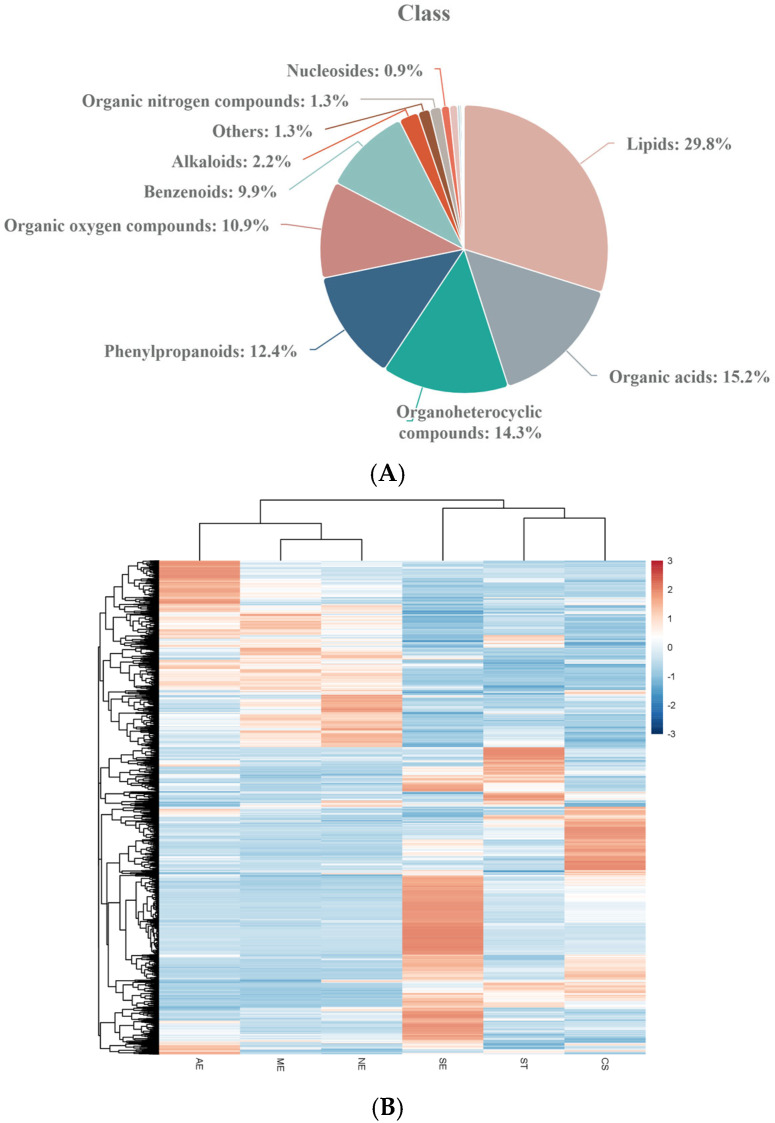
Analysis of differential metabolites in GE. (**A**) Classification pie chart; (**B**) hierarchical clustering heat map.

There were a total of 1783 differential metabolites in different parts of GE (Table S5, Figure 7A), which can be classified into 17 categories, mainly composed of lipids, organic acids, organoheterocyclic compounds, phenylpropanoids, benzenoids, and organic oxygen compounds. Studies have shown that polyphenols in GE can reduce the production of oxidative substrates by protecting mitochondrial function [34]. Organic acids can enhance the antioxidant defense ability of cells by regulating gene expression [35]. Organic acids can also inhibit the activity of *α*-amylase and *α*-glucosidase, delay the decomposition of complex carbohydrates and the release of glucose, and improve glucose metabolism by activating the AMPK pathway [36]. Phenylpropanoids can also exert hypoglycemic effects by inhibiting *α*-glucosidase, promoting GLP-1 secretion, enhancing peripheral glucose uptake, inhibiting liver gluconeogenesis, and activating the AMPK pathway [37]. Therefore, the differential metabolites in GE showed the characteristics of diversity, reflecting the complexity and diversity of GE in material metabolism and secondary metabolism.

To demonstrate the differences in the accumulation levels of differential metabolites in different parts of GE, HCA was conducted on the obtained differential metabolites. The relative quantitative values of the differential metabolites were scaled and clustered to construct a cluster heatmap, as shown in Figure 7B. The accumulation of 1783 metabolites in GE was affected by different parts and showed significant changes in the heat map. The six groups of samples can be roughly divided into two categories: the AE, ME, and NE were clustered into one category, and the other three groups were clustered into another category, indicating that the metabolites of different parts of GE were different. The metabolites of the underground part of GE were similar and can be clearly distinguished from the above-ground part. The content of some metabolites in the aerial part of GE was higher than that in the underground part. It could be seen that the non-medicinal part of GE also had certain medicinal development value, which provided a reference for its further development and utilization.

### 3.5. KEGG Annotation and Enrichment Analysis of Differential Metabolites

**Figure 8:**
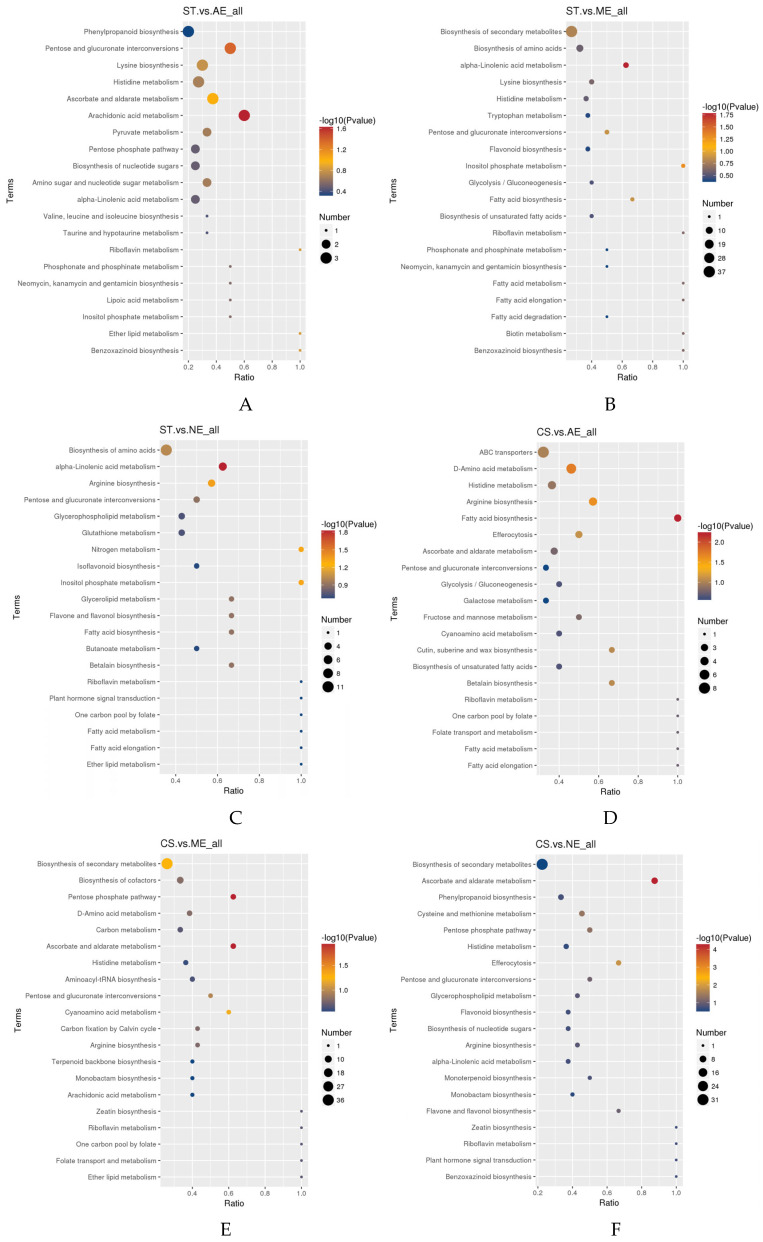

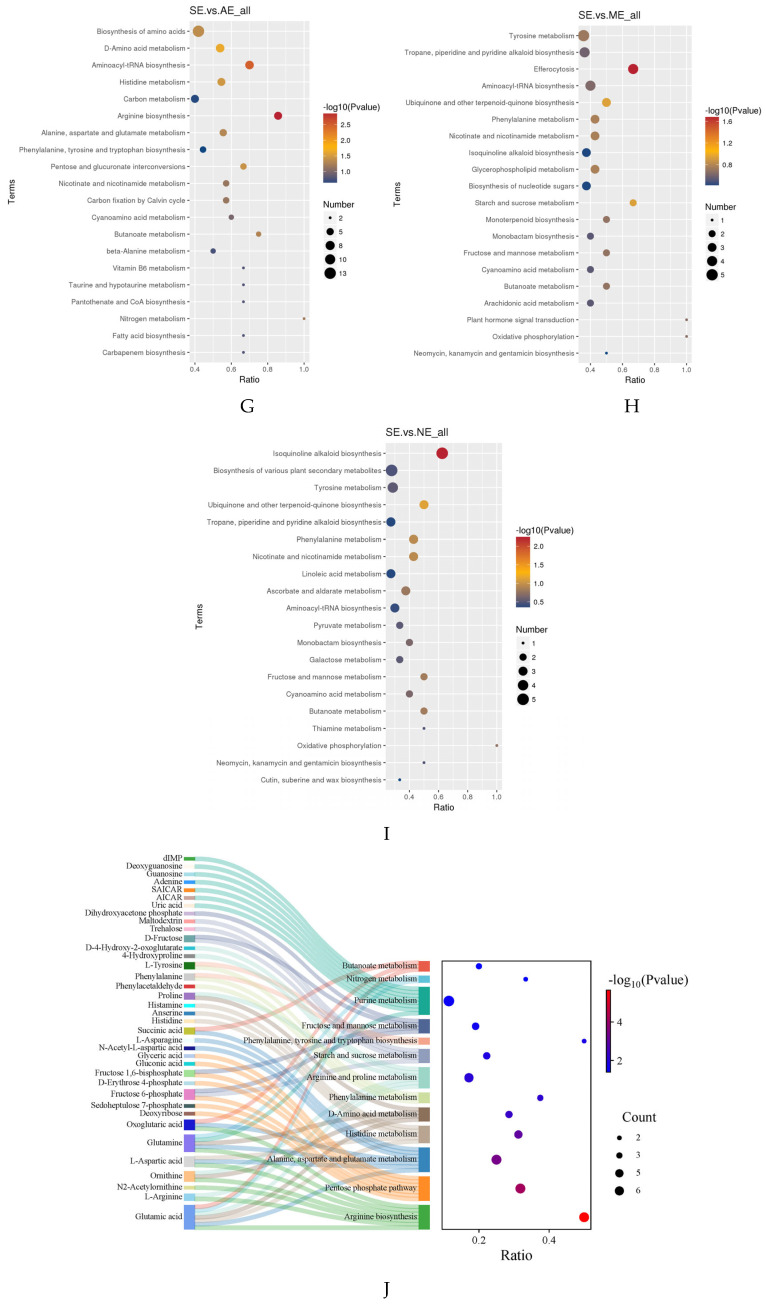
KEGG pathway bubble map of differential metabolites between the groups (**A**–**I**), mulberry base map of metabolites among the nine groups (**J**).

The KEGG database was utilized to annotate the differential metabolites in the comparison groups of different parts of GE, and the enrichment analysis of the annotation results was conducted. Among them, in the ST vs. AE group, a total of 38 differential metabolites were enriched in 34 pathways; in the ST vs. ME group, a total of 69 differential metabolites were enriched in 44 pathways; in the ST vs. NE group, a total of 55 differential metabolites were enriched in 59 pathways. In the CS vs. AE group, a total of 48 differential metabolites were enriched in 58 pathways; in the CS vs. ME group, a total of 61 differential metabolites were enriched in 50 pathways; in the CS vs. NE group, a total of 53 differential metabolites were enriched in 52 pathways. In the SE vs. AE group, a total of 73 differential metabolites were enriched in 59 pathways; in the SE vs. ME group, a total of 57 differential metabolites were enriched in 46 pathways; in the SE vs. NE group, a total of 56 differential metabolites were enriched in 42 pathways (Table S6). According to the *p*-value, the top 20 metabolic pathways with the highest significance were selected and represented by bubble diagrams (Figure 8A–I). The pathways that had a greater impact on the differences in each group included a variety of primary and secondary metabolic pathways, mainly involving flavonoid biosynthesis, biosynthesis of fatty acids, amino acids, and their derivatives. The active ingredients of traditional Chinese medicine exert extensive pharmacological effects at the molecular level through key nodes in these metabolic pathways, such as improving lipid metabolism, inhibiting tumors, antioxidation, and regulating immunity.

Pathway enrichment analysis was performed on the differential metabolites between the nine groups, and the differential metabolites enriched by each pathway were plotted using the bioinformatics platform (https://www.bioinformatics.com.cn, accessed on 20 February 2026) (Figure 8J). It was found that the differential metabolites in different parts of GE were mainly enriched in pathways such as arginine biosynthesis, pentose phosphate pathway, alanine, aspartate, and glutamate metabolism processes (Table S7). Among them, the difference in metabolites in different parts of GE may be related to the arginine biosynthesis pathway, mainly involving metabolites such as *L*-glutamate, *L*-arginine, N-acetylornithine, *L*-ornithine, *L*-aspartate, *L*-glutamine, and oxoglutaric acid. It was worth noting that the relative content of *L*-aspartate in CS was higher than that in AE and NE, and the relative content of *L*-arginine was higher than that in ME and NE, suggesting that CS may be the main part for the synthesis of *L*-arginine and *L*-aspartate. These results suggest that the dynamic changes in amino acid metabolites may reflect the different needs of antioxidant or defensive metabolism in different organs. These findings provided a theoretical basis for the in-depth analysis of the tissue-specific synthesis mechanism of amino acid compounds in GE and also laid a foundation for the targeted development and utilization of different parts in GE.

### 3.6. Bioactivity Evaluation in Different Parts of GE

#### 3.6.1. Antioxidant Activity Evaluation In Vitro

Oxidative stress is associated with the pathogenesis of various diseases [38,39]. In order to comprehensively evaluate the antioxidant potential of different parts of the GE samples, we employed two widely used chemical analysis methods, DPPH and ABTS, to compare their free radical scavenging ability with that of VC in order to assess the overall antioxidant capacity of GE. The results are shown in Table 2. The IC_50_ value is inversely proportional to the antioxidant capacity, and the larger the value, the weaker the antioxidant capacity. The results showed that there were significant differences in the ability of samples from different parts of GE to scavenge DPPH and ABTS free radicals. It is worth noting that the scavenging ability of ST to DPPH free radical was stronger than that of SE, AE, and CS. However, in the ABTS free radical scavenging test, the scavenging activity of CS was the highest, followed by SE and ST, which may be due to the different mechanisms of the ABTS and DPPH reactions. The results of in vitro antioxidant activity showed that the free radical scavenging ability of the above-ground parts, such as ST, CS, and SE of GE, was stronger than that of underground tubers. This result may be related to the differences in metabolites in different parts of plants, which produce different levels of antioxidant components due to their specific functions in plant metabolism and defense. In general, the above-ground parts of plants are susceptible to biotic or abiotic stresses, such as light, pests, herbivores, etc., and plants need to produce a large number of antioxidant metabolites to prevent and resist stress [40]. This is consistent with the results of this study. As far as the tuber of GE is concerned, the antioxidant results obtained by the two detection methods were consistent, and the order of scavenging ability against DPPH and ABTS free radicals was AE, ME, and NE. The concentration range of the samples, goodness of fit (R^2^), and confidence interval (95% CI) in the DPPH and ABTS free radical scavenging experiments are shown in Table S8.

#### 3.6.2. Evaluation of α-Glucosidase Inhibitory Activity

GE is often used as a medicinal material in hypoglycemic prescriptions in traditional Chinese medicine. This study compared the inhibitory activity on *α*-glucosidase in different parts of GE, which was of great significance for the study on the material basis of *α*-glucosidase inhibitory activity and development and utilization of GE. The results of Table 3 showed that there were significant differences in the inhibitory activity against *α*-glucosidase in different parts of GE, and the *α*-glucosidase inhibitory activity of six groups of samples was weaker than that of acarbose. The inhibitory activity of ST, AE, and CS was stronger than that of SE, ME, and NE. This indicated that the above-ground parts of GE also had potential *α*-glucosidase inhibitory activity. The inhibitory activity against *α*-glucosidase is not determined by a single component but is the result of the synergistic effect of multiple compounds. Unlike relatively stable tubers (mainly serving as the storage organs for nutrients), ST and CS, as reproductive organs, have more active metabolic activities and are likely to synthesize some highly concentrated and structurally unique active components. In addition, the uneven distribution of active ingredients within tubers also makes the *α*-glucosidase inhibitory potential of ST and CS more prominent in comparison with tubers. The concentration range, goodness of fit (R^2^), and confidence interval (95% CI) of the samples in the *α*-glucosidase inhibition test are shown in Table S8.

#### 3.6.3. Correlation Analysis Between Biological Activity and Key Metabolites of GE

**Figure 9:**
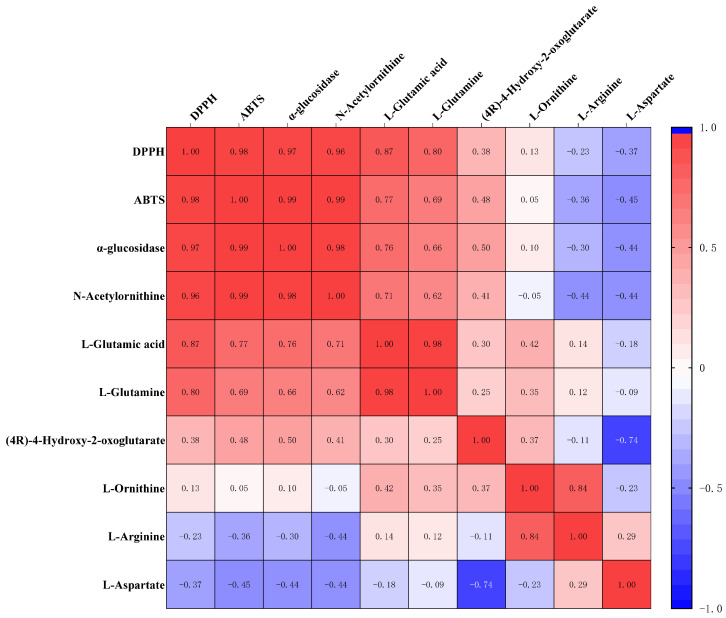
Heat map of the correlation between key differential metabolites and IC_50_ values. Different letters above columns and the color of the column indicate the correlation, with the larger and redder the color, the greater the correlation coefficient.

In order to reveal the relationship between the biological activities of GE and the seven differential metabolites in the arginine biosynthesis pathway, we calculated the correlation coefficients between the differential metabolite data of different parts from GE and the IC_50_ values of the inhibitory activities to DPPH, ABTS free radicals, and *α*-glucosidase of the samples from each part using the Pearson method. The analysis was conducted using the average values of each variable, and the correlation heatmap is shown in Figure 9. The IC_50_ values of the antioxidant and *α*-glucosidase inhibitory activity of GE were negatively correlated with *L*-arginine and *L*-aspartate. That is to say, the lower IC_50_ values of GE for anti-oxidation and inhibition of *α*-glucosidase were associated with higher relative levels of *L*-arginine and *L*-aspartate. It is preliminarily inferred that *L*-arginine and *L*-aspartate may be involved in the antioxidant and *α*-glucosidase inhibition process of GE. According to the box plot (Figure S5) of relative quantitative analysis of differential metabolites, it could be seen that the relative content of metabolites positively correlated with biological activity in the above-ground part was higher, while the relative content of negatively correlated substances in the tubers was higher than that in the above-ground part, preliminarily indicating that the above-ground part of GE also has exploitable value. The utilization of the above-ground resources is an effective way to alleviate the shortage of GE resources and improve the utilization rate of GE plants, which is conducive to the development and sustainable development of non-traditional medicinal parts of Chinese herbal medicines.

## 4. Discussion

According to the distribution of metabolites in different parts of GE and the results of PCAs, the metabolic profile characteristics of the above-ground and underground tubers of GE were clearly distinct. The fundamental reason lies in the difference in functional division and environmental adaptability of each part. In general, the above-ground parts of plants are directly exposed to sunlight, and a large amount of flavonoids, anthocyanins, and other substances need to be synthesized to resist photooxidative stress [41]. The roots often accumulate metabolites with specific antibacterial activity, such as phenolic acids and saponins, to defend against the risk of pathogenic fungi in the soil, or adjust the composition of metabolites to adapt to drought stress. In addition, the underground parts absorb water and minerals from the soil and store metabolites transported from the above-ground parts or synthesized by themselves [42]. More than 90% of the life cycle of GE is underground, relying on symbiosis with Armillaria to obtain nutrition. When sufficient nutrients have been accumulated in the tuber, bolting and flowering occur, and the plant transitions into the reproductive growth stage. The study found that during the bolting process of GE, amino acids and their derivatives, alkaloids, and other substances in the tubers were significantly up-regulated, providing nitrogen sources and signal molecules for plant growth, while energy storage substances such as fatty acids and organic acids were significantly down-regulated [12]. This unique growth pattern determines the metabolic differences between the above-ground and underground parts.

In order to clarify the mechanism of the metabolic differences between the above-ground and underground parts of GE, we set up nine comparison groups and conducted KEGG enrichment analysis. The differential metabolites in different comparison groups of GE mainly involved pathways such as the biosynthesis of flavonoids, the biosynthesis of fatty acids, and amino acids and their derivatives. Flavonoids are an important class of active substances in GE, which have strong antioxidant, antibacterial, anti-allergic, anti-tumor, and other pharmacological activities. The biosynthesis of flavonoids starts from the phenylpropanoid metabolic pathway, which is one of the core pathways of plant secondary metabolism [43]. The anti-inflammatory and antioxidant activities of flavonoids are closely related to the number and position of phenolic hydroxyl groups in their chemical structures. Studies have shown that the strong free radical scavenging ability of flavonoids in Codonopsis Radix is mainly achieved by inhibiting the NF-κB pathway and activating the Nrf2/ARE pathway [44]. The differences in gene expression of the fatty acid synthesis pathway not only affect the accumulation of metabolites in plants themselves but also lead to differences in the efficacy of different origin herbs. The combined analysis of metabolomics and transcriptomics revealed that the differentially expressed genes between Bupleurum scorzonerifolium and Bupleurum chinensis DC were mainly enriched in the biosynthesis of unsaturated fatty acids and the fatty acid metabolism pathway, providing a theoretical basis for the stronger anti-inflammatory and liver-protective effect of Bupleurum chinensis DC and the better antipyretic effect of Bupleurum scorzonerifolium [45]. Through quantitative proteomics and targeted amino acid metabolomics, Liu et al. [46] found that the functions of differentially expressed proteins after parthenolide treatment of lung cancer cells were enriched in amino acid metabolism and oxidative stress-related pathways. Yunsheng Wang et al. [47] detected a total of 345 metabolites in *Gastrodia elata*. The gene-metabolite association analysis identified 16 pairs of differentially expressed genes that were significantly correlated with the differentially accumulated metabolites, involving the amino acid biosynthesis pathway, revealing the nutritional basis of GE as a “medicine and food homologous’’ substance. In this study, the total differential metabolites from different parts of GE were significantly enriched in the arginine biosynthesis pathway. In this pathway, the main metabolites involved include *L*-glutamic acid, *L*-arginine, N-acetylornithine, *L*-ornithine, *L*-aspartate, *L*-glutamine, oxoglutaric acid, etc. A number of studies have shown that traditional Chinese medicine or active molecules can improve diseases by regulating a variety of related targets and metabolites in the arginine biosynthesis pathway [48,49]. An amino acid is the basic unit of protein, which is directly involved in every link from germination to maturity of traditional Chinese medicine resources. Exogenous supplementation of amino acids can directly promote the growth of medicinal plants and the accumulation of active ingredients, thereby influencing their quality and efficacy [50].

The results of in vitro activity experiments on GE showed that the antioxidant capacity of the above-ground part of GE was higher than that of the underground tuber. The inhibitory activity of ST and CS on *α*-glucosidase was stronger than that of SE, ME, and NE, especially the inhibitory activity of ST, which was the most significant. To further explore the metabolites related to the biological activity of GE, we conducted a correlation analysis. The results showed that the antioxidant and *α*-glucosidase inhibitory abilities of GE were positively correlated with *L*-arginine and *L*-aspartate, indicating that *L*-arginine and *L*-aspartate are metabolites preliminarily associated with the antioxidant and *α*-glucosidase inhibitory effects of GE. The antioxidant and hypoglycemic mechanisms of different amino acids are different. Research has shown that arginine may enhance the body’s antioxidant defense ability by activating the Nrf2 signaling pathway and promoting GSH synthesis, and directly improve insulin resistance by increasing the activity of antioxidant enzymes and reducing the release of inflammatory factors [51,52]. Aspartate promotes the generation of antioxidant metabolites by significantly altering the microbial community structure and can efficiently synergize with glucose to amplify insulin release signals when blood glucose levels rise [53,54]. However, the above mechanism is speculative, which has not been experimentally verified in this study, and its mechanism needs to be further verified.

In addition, we also found that *L*-arginine and *L*-aspartate had different degrees of positive or negative relationships with other amino acids, and *L*-glutamic acid, *L*-ornithine, *L*-glutamine, etc., were negatively correlated with the biological activity of GE. The function of amino acids in the body depends on whether they are key substrates for energy metabolism or are abnormally accumulated in pathological conditions. For instance, the traditional Chinese medicine compound Xiaozhang Tie significantly increased the arginine level and simultaneously decreased the serum NO level, thereby alleviating the gastrointestinal motility disorder in rats with liver cirrhosis [55]. Methionine can be converted into cysteine through the trans-sulfur pathway, and cysteine, as the limiting substrate for glutathione synthesis, directly affects the body’s ability to eliminate reactive oxygen species [56]. Metabolomics analysis revealed that after treatment with Yougui Pill, the levels of seven metabolites, such as isoleucine and valine, in the allergic asthma model mice induced by dust mites were significantly decreased [57]. It can be seen that different amino acids bear complex and diverse functions in organisms and are dynamically changing. The efficacy of traditional Chinese medicine is the result of synergy between various active ingredients. Therefore, the biological activity characteristics of GE are inseparable from its unique metabolites.

There are still some limitations in this study. Firstly, the samples of GE used in this research were collected only from a single location in China and were collected only once, which failed to reflect the effects of different harvesting periods and different ecological environments on the chemical composition of GE. Secondly, the biological activity data of GE in this study were from in vitro experiments, which cannot fully simulate the complex pharmacokinetic process in vivo. Therefore, the effective concentration and effect in vitro need to be further verified by animal experiments and clinical studies. Thirdly, the sample size of the correlation analysis between the biological activity of GE and key metabolites was small, and the results were susceptible to outliers, which may lead to insufficient statistical power and less robust correlation coefficients. Therefore, the current metabolite-activity association analysis was still a preliminary exploration, which needs to be further verified by large samples. Fourthly, only samples of GE produced in China were used in this study, and it is not clear whether the conclusion is applicable to germplasm resources from other regions (such as South Korea, Japan, etc.). Finally, the tubers of GE were steamed at 100 degrees Celsius, while other plant parts were directly dried. Although the purpose of steaming was to inactivate enzymes and preserve active ingredients, we cannot completely rule out the possibility that the differences between underground and above-ground tissues were caused by different pre-treatment methods. The rationality of these different treatment methods will be further verified.

## 5. Conclusions

In summary, in this study, UHPLC-Q Exactive Orbitrap-MS combined with multivariate statistical analysis and in vitro biological activity determination methods were used to evaluate the metabolites and activity differences in different parts of GE, and it was found that their distribution was closely related to organ-specific functions. Various types of compounds, such as lipids and organic acids, were identified from the extracts of different parts of GE. PCA and OPLS-DA analyses indicated that there were significant differences in the metabolic characteristics of the aerial parts and tubers of GE. Based on VIP values, FC values, and *p*-values, differential metabolites were screened, and KEGG enrichment analysis was conducted, preliminarily revealing that the differences in different parts of GE were related to the arginine biosynthesis pathway. In the evaluations of the activities at different parts, the above-ground parts of GE also possessed good antioxidant and *α*-glucosidase inhibitory potentials. *L*-arginine and *L*-aspartate may be associated with the antioxidant and *α*-glucosidase inhibitory effects of GE. Therefore, the non-medicinal parts of GE can be regarded as potential medicinal resources. These findings provide a theoretical basis for in-depth analysis of the tissue-specific synthesis mechanism of the compounds in GE and also offer a foundation for analyzing the medicinal value of different parts of GE and its development and utilization, as well as reducing the waste of medicinal resources. Further research is needed to clarify the molecular mechanisms by which these metabolites exert antioxidant and *α*-glucosidase inhibitory effects.

## Figures and Tables

**Table 1:** Statistics of metabolites identified by GE.

Primary Classification	Secondary Classification	Number of Metabolites
Lipids	Prenol lipids	268
	Fatty Acyls	262
	Steroids and steroid derivatives	78
	Glycerophospholipids	64
	Glycerolipids	24
	Others	18
Organic acids	Carboxylic acids and derivatives	308
	Hydroxy acids and derivatives	22
	Keto acids and derivatives	20
	Others	43
Organoheterocyclic compounds	Indoles and derivatives	33
	Benzopyrans	30
	Quinolines and derivatives	25
	Lactones	20
	Others	247
Phenylpropanoids	Flavonoids	91
	Cinnamic acids and derivatives	35
	Coumarins and derivatives	32
	Others	136
Benzenoids	Benzene and substituted derivatives	137
	Phenols	58
	Naphthalenes	21
	Others	46
Organic oxygen compounds	Organooxygen compounds	249
	Others	2
Others		203

**Table 2:** Antioxidant activity in different parts of GE.

Sample	IC_50_ (mg/mL)	
	DPPH	ABTS
ST	7.68 ± 0.52 ^d^	85.96 ± 3.60 ^d^
CS	27.59 ± 4.32 ^c^	61.71 ± 3.35 ^e^
SE	24.32 ± 2.42 ^c^	82.81 ± 1.82 ^d^
AE	27.09 ± 3.38 ^c^	96.96 ± 1.62 ^c^
ME	94.24 ± 4.71 ^b^	577.90 ± 3.10 ^b^
NE	166.60 ± 4.68 ^a^	795.30 ± 2.19 ^a^
VC	0.01 ± 0.00 ^d^	0.16 ± 0.01 ^f^

Note: IC_50_ values were presented as the mean value ± SD of three independent experiments (*n* = 3). Different letters within the same column indicated significant differences, *p* < 0.05.

**Table 3:** *α*-glucosidase inhibitory activity in different parts of GE.

Sample	IC_50_ (mg/mL)
ST	5.44 ± 0.29 ^d^
CS	9.90 ± 0.49 ^d^
SE	19.86 ± 4.16 ^c^
AE	6.89 ± 0.46 ^d^
ME	134.47 ± 8.28 ^b^
NE	171.47 ± 3.77 ^a^
acarbose	0.02 ± 0.00 ^e^

Note: IC_50_ values were presented as the mean value ± SD of three independent experiments (*n* = 3). Different letters within the same column indicated significant differences, *p* < 0.05.

## Data Availability

The raw metabolomic data generated by UHPLC-MS/MS in this study, including feature matrix, metabolite annotation files, processing parameters, and analysis outputs, are available from the corresponding author upon reasonable request.

## Supplementary Materials

The following supporting information can be downloaded at: https://www.mdpi.com/article/10.3390/metabo16060406/s1, Figure S1: QC sample monitoring results. (A) PCA diagram; (B) Correlation analysis chart; Figure S2: Total ion chromatogram (TIC) of QC sample. (A) Positive ion mode; (B) Negative ion mode; Figure S3: OPLS-DA validation charts, ST vs. AE, ST vs. ME, ST vs. NE, CS vs. AE, CS vs. ME, CS vs. NE, SE vs. AE, SE vs. ME, and SE vs. NE (A–I). The horizontal axis represents the retention degree of permutations in the permutation test, and the vertical axis represents the R^2^Y or Q^2^ values obtained from the permutation test. The two dotted lines respectively represent the regression lines of R^2^Y and Q^2^; Figure S4: The number of differential metabolites in different comparison groups. Red columns and blue columns represented up-regulated and down-regulated differential metabolites, respectively; Figure S5: Box plot of the contents of key differential metabolites in GE; Table S1: Lists before and after deleting compounds with a coefficient of variation (CV) greater than 30%; Table S2: List and characteristics of the metabolites identified in GE; Table S3: Relative contents of metabolites in different parts of GE (%); Table S4: The statistical parameters obtained by the OPLS-DA model; Table S5: List of differential metabolites in GE; Table S6: List of enriched KEGG pathways in different groups; Table S7: List of enriched KEGG pathways by total differential metabolites; Table S8: IC_50_ values and fitting parameters.

## Abbreviations

The following abbreviations are used in this manuscript:

GE	*Gastrodia elata* Blume
ST	stem
CS	capsule shell
SE	seed
AE	arrow end
ME	middle end
NE	navel end
KEGG	Kyoto Encyclopedia of Genes and Genomes
PCA	principal component analysis
OPLS-DA	orthogonal partial least squares discriminant analysis
QC	quality control
HCA	hierarchical clustering analysis
VIP	variable importance in the projection
FC	fold change
PBS	phosphate-buffered saline
SD	standard deviation
IC_50_	half maximal inhibitory concentration
