# Supplementary material for: Comparative Analysis of Components and Biological Activities in Different Parts of Gastrodia elata Blume
Source: Metabolites. 2026 Jun 10;16(6):406. doi: 10.3390/metabo16060406 (PMC13303185; doi:10.3390/metabo16060406)
Supplement: Supplementary file 1 [file metabolites-16-00406-s001.zip › supplementary figures.pdf]

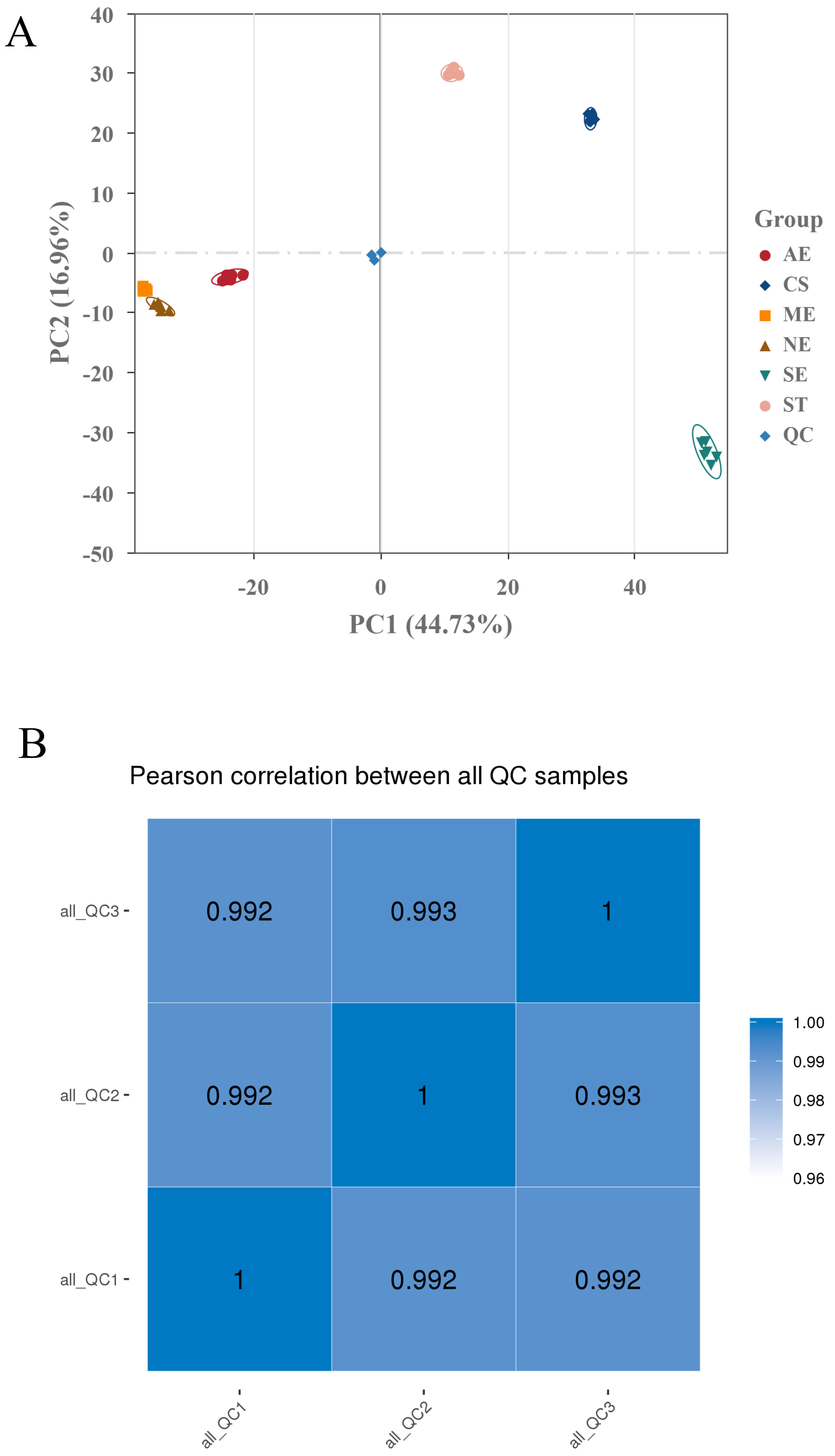

Figure S1. QC sample monitoring results. (A) PCA diagram; (B) Correlation analysis chart

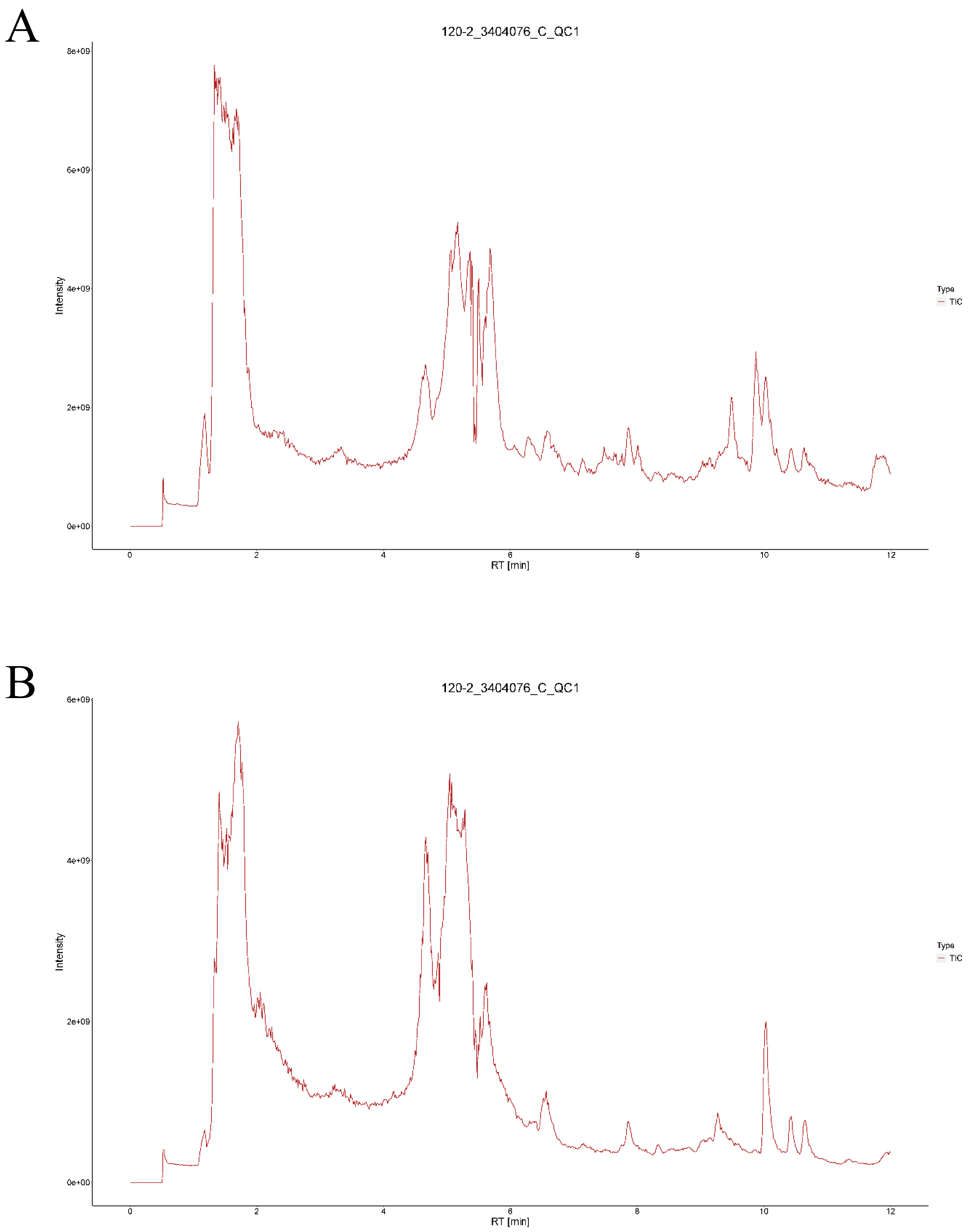

Figure S2. Total ion chromatogram (TIC) of QC sample. (A) Positive ion mode; (B) Negative ion mode

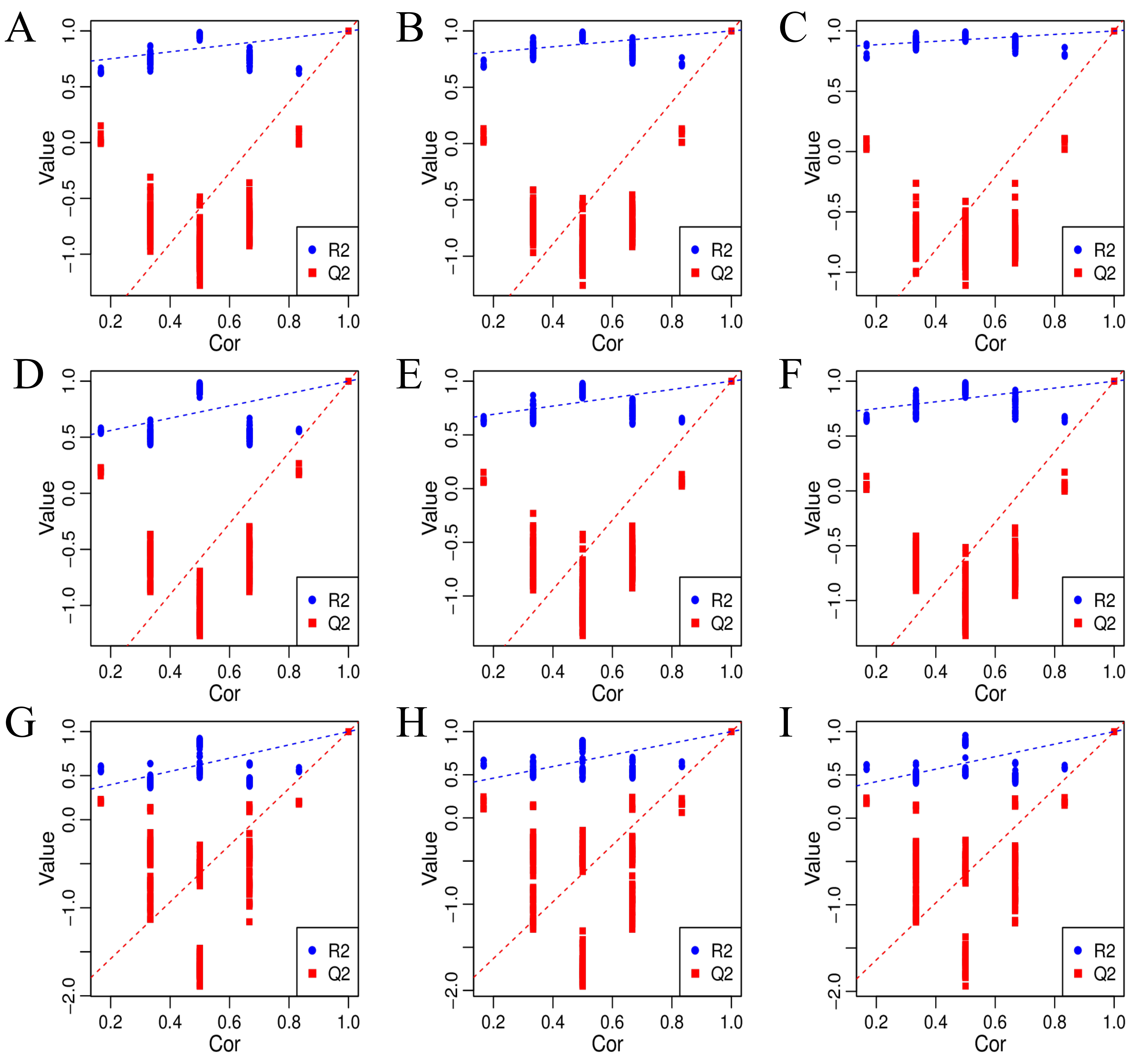

Figure S3. OPLS-DA validation charts, ST vs AE, ST vs ME, ST vs NE, CS vs AE, CS vs ME, CS vs NE, SE vs AE, SE vs ME, and SE vs NE (A-I). The horizontal axis represents the retention degree of permutations in the permutation test, the vertical axis represents the R^2Y or Q^2 values obtained from the permutation test. The two dotted lines respectively represent the regression lines of R^2Y and Q^2.

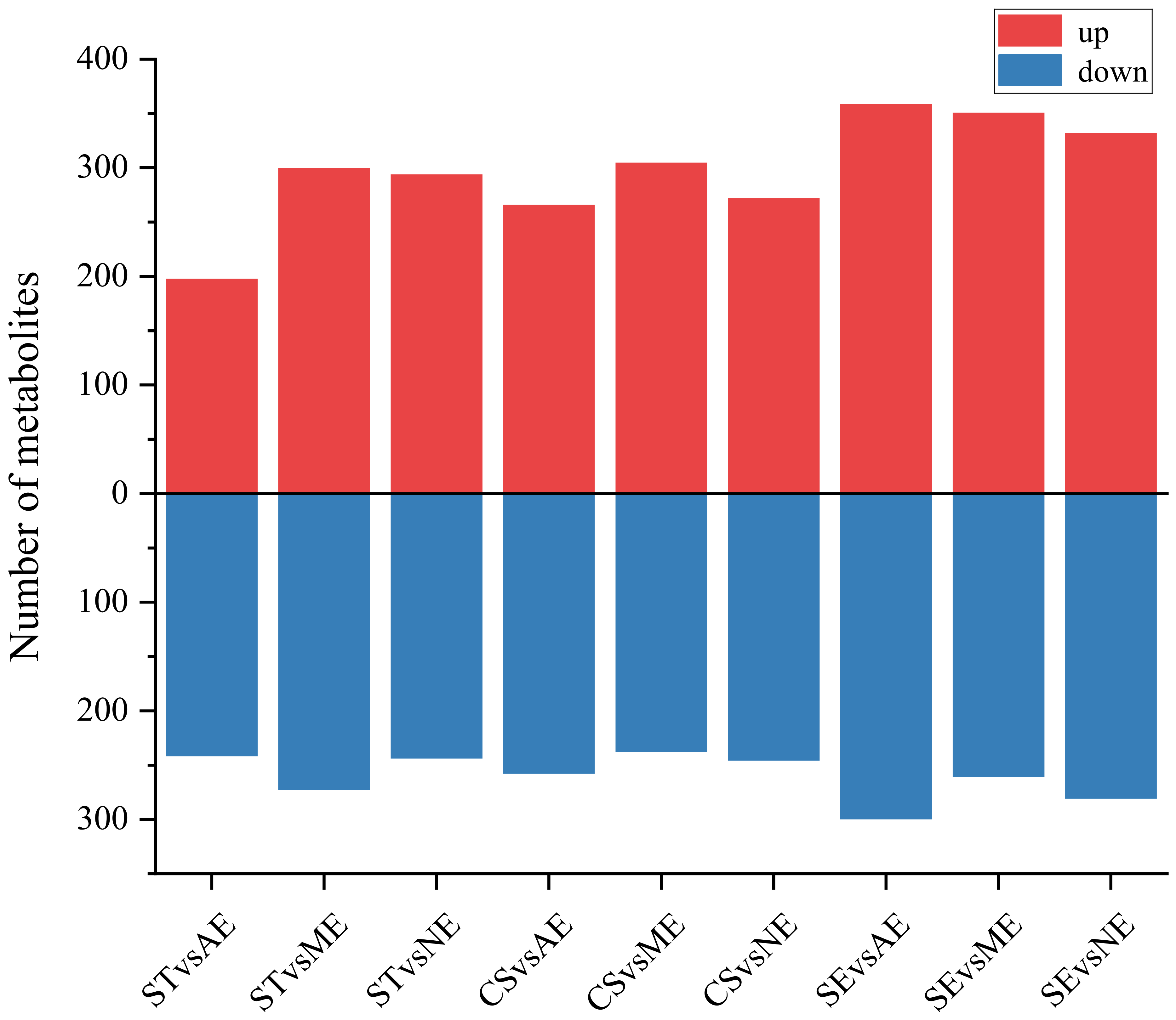

Figure S4. The number of differential metabolites in different comparison groups. Red columns and blue columns represented up-regulated and down-regulated differential metabolites, respectively.

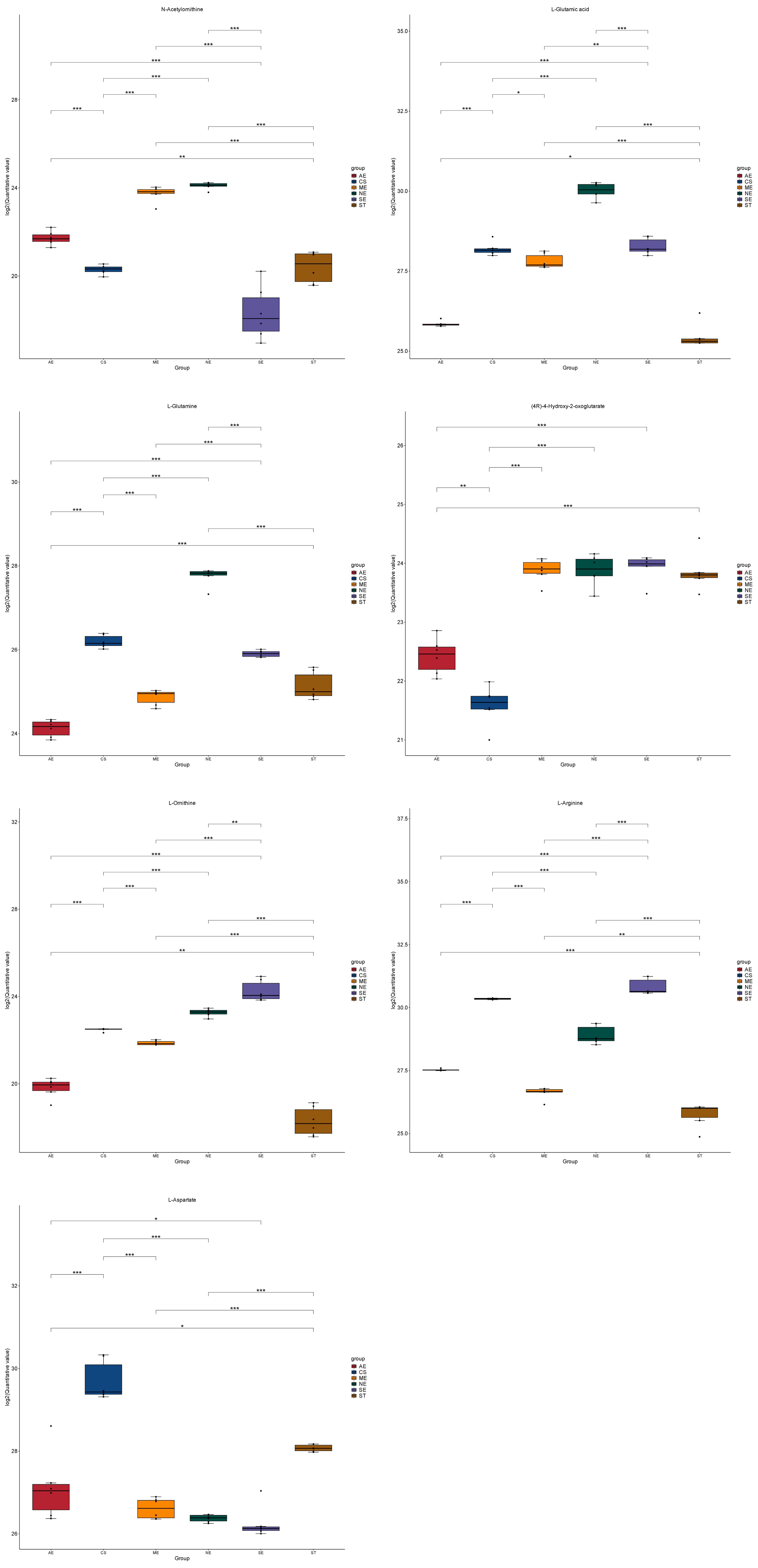

Figure S5. Box plot of the contents of key differential metabolites in GE.
